# Resistance to Antimicrobial Peptides in Vibrios

**DOI:** 10.3390/antibiotics3040540

**Published:** 2014-10-27

**Authors:** Delphine Destoumieux-Garzón, Marylise Duperthuy, Audrey Sophie Vanhove, Paulina Schmitt, Sun Nyunt Wai

**Affiliations:** 1 Ecology of Coastal Marine Systems, CNRS, Ifremer, University of Montpellier, IRD, Place Eugène Bataillon, CC80, 34095 Montpellier, France; E-Mail: avanhove@univ-montp1.fr; 2 Department of Molecular Biology, The Laboratory for Molecular Infection Medicine Sweden (MIMS), Umeå University, 901 87 Umeå, Sweden; E-Mails: marylise.duperthuy@molbiol.umu.se (M.D.); sun.nyunt.wai@molbiol.umu.se (S.N.W.); 3 Laboratorio de Genética e Inmunología Molecular, Instituto de Biología, Pontificia Universidad Católica de Valparaíso, Avenida Universidad 330, 2373223 Valparaíso, Chile; E-Mail: paulina.schmitt@ucv.cl

**Keywords:** vibrio, lipopolysaccharide, outer membrane vesicle, membrane transporter, innate immunity, defensin, cathelicidin, bactericidal/permeability-increasing protein

## Abstract

Vibrios are associated with a broad diversity of hosts that produce antimicrobial peptides (AMPs) as part of their defense against microbial infections. In particular, vibrios colonize epithelia, which function as protective barriers and express AMPs as a first line of chemical defense against pathogens. Recent studies have shown they can also colonize phagocytes, key components of the animal immune system. Phagocytes infiltrate infected tissues and use AMPs to kill the phagocytosed microorganisms intracellularly, or deliver their antimicrobial content extracellularly to circumvent tissue infection. We review here the mechanisms by which vibrios have evolved the capacity to evade or resist the potent antimicrobial defenses of the immune cells or tissues they colonize. Among their strategies to resist killing by AMPs, primarily vibrios use membrane remodeling mechanisms. In particular, some highly resistant strains substitute hexaacylated Lipid A with a diglycine residue to reduce their negative surface charge, thereby lowering their electrostatic interactions with cationic AMPs. As a response to envelope stress, which can be induced by membrane-active agents including AMPs, vibrios also release outer membrane vesicles to create a protective membranous shield that traps extracellular AMPs and prevents interaction of the peptides with their own membranes. Finally, once AMPs have breached the bacterial membrane barriers, vibrios use RND efflux pumps, similar to those of other species, to transport AMPs out of their cytoplasmic space.

## 1. Introduction

Vibrios are γ-proteo-bacteria ubiquitous in aquatic environments. They have evolved the capacity to colonize a broad series of hosts from protozoans to metazoans. Vibrios are normally present in the tissues of healthy animals. Sometimes they become pathogenic in wild marine animals such as corals, in particular as a result of environmental changes including shifts in seawater temperature and salinity, or, for aquacultured animals, upon exposure to high animal densities or stressful farming practices [1]. Currently, vibrioses are recognized as a major factor limiting the development of aquaculture. In addition, vibrios can cause severe disease outbreaks in human populations, the best known example being cholera. Again, environmental drivers—such as temperature changes, severe rainfalls that lower water salinity, and insufficient sanitation—govern the occurrence of the disease in human populations [2].

Vibrios have developed tropism for epithelial tissues that line both the outside and inside of cavities and lumen of their diverse hosts. They can colonize the keratinized epithelium of skin as well as the gastrointestinal tract. By lining the cavities and surfaces of structures throughout the body, epithelia act as a first line of defense against pathogens. Epithelia also produce antimicrobial peptides (AMPs) [3,4], conferring to the host an immune arsenal broadly conserved among metazoans. When the host's epithelial barriers are breached, vibrios encounter phagocytes, key components of the animal immune system. These phagocytes infiltrate infected tissues and use reactive oxygen and nitrogen species (ROS and RNS) as well as AMPs to kill phagocytosed microorganisms intracellularly or deliver their antimicrobial content extracellularly to circumvent infection. Interestingly, recent works have shown that vibrios are able to colonize and survive inside phagocytes [5,6].

AMPs from metazoans are often cationic peptides that initially interact electrostatically with the membranes of bacteria, which carry negatively-charged lipopolysaccharide (Gram-negative) or teichoic acids (Gram-positive). Many AMPs then insert into the membranes and form deleterious pores or channels [7]. Alternatively, AMPs can bind to essential components of bacterial membranes or translocate across to reach the cytoplasm, where they interfere with essential cellular processes such as nucleic acid, protein, enzyme, and cell wall syntheses [8,9,10,11,12,13]. In addition, AMPs produced by a given host can be synergistic, combining their mechanisms of action to fight bacterial pathogens [14]. However, it has become clear that the activity of AMPs goes far beyond their antimicrobial properties; these peptides are also involved in many immunomodulatory functions including inflammation, wound healing, chemotaxis, cell differentiation, angiogenesis, regulation of oxidative stress, regulation of adaptive immunity, and epithelia homeostasis [4,15]. Accordingly, AMPs are also called Host Defence Peptides.

Importantly, the tissues of healthy metazoans host an abundant microbiota, which itself has the capacity to produce AMPs, contributing to protection against pathogenic microbes. Prokaryotic AMPs are frequently referred to as bacteriocins, a generic name that covers classes of compounds with diverse structures and mechanisms of action. Bacteriocins may be peptides created by complex biosynthetic pathways that enable the inclusion of unconventional amino acids, as well as nucleotides and siderophores [16,17]. Many of these prokaryotic AMPs are cationic, although this is not a general rule. Like metazoan AMPs, some target the bacterial membranes while others target specific receptors and behave as inhibitors of key metabolic pathways. Still others combine different mechanisms of action (for review see [17]).

When confronted with such a complex immune arsenal, how do vibrios avoid the chemical defenses of their hosts and associated microbiota? What can we learn from their ability to colonize immune cells/tissues that produce high local concentrations of AMPs? In the context of the extensive antibiotic use that has led to emergence of broad-spectrum antibiotic-resistant bacteria [18], AMPs are often seen as an alternative to conventional antibiotics. However, an increasing number of studies have shown the diversity of mechanisms by which bacteria can also avoid the action of AMPs. Thus, the emergence of “superbugs” resistant to both antibiotics and AMPs is a potential risk of using AMPs as an antibiotic alternative. However, understanding the mechanism by which bacteria have evolved the capacity to live in AMP-producing tissues should allow us to develop strategies to prevent AMP-resistance.

## 2. Antimicrobial Peptides in Host-Vibrio Interactions

### 2.1. Vibrios Colonizing Epithelial Surfaces

Many species of vibrios pathogenic for human and animal species have evolved the capacity to colonize epithelia (Table 1). Among these, the species of vibrios pathogenic for humans, *Vibrio* *cholerae* and *Vibrio parahaemolyticus,* cause major enteric disorders. While *V. parahaemolyticus* disrupts the intestinal epithelium [19], *V. cholerae* induces inflammatory responses and innate immune cell infiltration in the small intestine without affecting the integrity of the mucosal tissue [20]. Diarrhea caused by such enteric infections leads to intense dehydration and is recognized as a major factor in morbidity and mortality worldwide. Virulence factors of the diarrheagenic vibrios are expressed upon intimate association with host epithelial cells and, in many instances, include the secretion of toxins. Vibrios causing gastrointestinal infection need to penetrate the mucous layer before attaching to intestinal epithelial cells, a process usually mediated by fimbriae or pilus structures (e.g., toxin-co-regulated pilus (TCP)). Subsequently, the bacteria secrete important virulence factors such as cholera toxin (CT) and hemagglutinin/protease (HA/protease) (for review see [21]). In *V. parahaemolyticus*, colonization of the intestine is dependent on the type III secretion system (T3SS2) [22] and further secretion of a T3SS2-secreted effector, VopZ, which also inhibits host mucosal defenses [23].

As in humans, many vibrios colonize the epithelial surfaces of animals, both vertebrates and invertebrates. Again, this often requires a first step of binding to the mucus covering the epithelium. In some cases, epithelium colonization is part of a mutualistic process. For instance, in the squid, the luminescent *Vibrio fischeri* colonizes the crypts of the squid light organ, which consists of a series of deep invaginated epithelium-lined crypt spaces [24]. In other cases, invasion of the epithelium is part of the pathogenic process. For instance, in the rainbow trout, *Vibrio anguillarum* colonizes both the skin and the intestinal epithelia, causing a fatal hemorrhagic septicaemia [25]. Similarly, in the coral *Oculina Patagonica*, the pathogenic *Vibrio shiloi* penetrates into the epithelial cells of the coral, multiplies, and produces a toxin that inhibits photosynthesis of the coral symbiotic algae (for review see [26]).

**Table 1:** Vibrios colonizing epithelia.

Species or strain	Host	Tissues	References
*V. cholerae*	human	intestine	[20]
*V. vulnificus*	human	skin, wounds	[27]
*V. parahemolyticus*	human	intestine	[19]
*V. anguillarum*	fish	skin, intestine	[25]
*V. shiloi*	coral	oral ectoderm	[26]
*V. coralliilyticus*	coral	oral ectoderm	[28]
*V. fisheri*	squid	light organ	[24]

### 2.2. AMPs and Epithelial Defenses

Mammalian epithelial tissues such as the epidermis but also the respiratory, gastrointestinal and genitourinary tracts are in direct contact with the environment, thus, constant interaction between microorganisms and the immune system occurs at these sites. In vertebrates, epithelial tissues provide the first line of protection as they trigger the immune response. Mammalian epithelial cells function as both a physical barrier and as immune active cells, producing a number of immune-related molecules [29]. Therefore, colonizing vibrios face a diversity of chemical weapons expressed in epithelial tissues. Indeed, in animals, virtually all epithelia have been found to express AMPs either constitutively or in response to damage and/or infection (Table 2).

***In humans***, AMPs are expressed in a broad range of epithelial cell types, either constitutively or in response to infection. The major AMPs and proteins of human epithelia include the small cationic α- and β-defensins, the human cathelicidin LL-37 (hCAP-18) and the bactericidal/permeability-increasing protein (BPI). The average concentration of defensins in these epithelial cells reaches the 10–100 µg/mL range with higher local concentrations due to the uneven distribution of defensins [3]. BPI is expressed in mucosal epithelia including the esophagus and the colon [30]. LL-37 is expressed in the squamous epithelia of the airways, mouth, tongue, esophagus and large intestine [31,32,33] as well as in inflamed skin [34]. Human β-defensins are expressed by kidney, skin, pancreas, gingiva, tongue, esophagus, salivary gland, cornea, and airway epithelium [35]. In the small intestine, the antimicrobial C-type lectins HIP/PAP are expressed [4,36] together with enteric α-defensins, which are major AMPs exclusively expressed by Paneth cells located at the bottom of the intestinal crypts [37]. Importantly, the epithelial lining of the small intestine is the site at which *V. cholerae* adheres after passing through the gastric acid barrier and penetrating the mucin layer of the small intestine [38].

The human enteric α-defensins HD-5 and HD-6 are components of the secretory granules of Paneth cells. They are released in the lumen of the small intestinal crypts upon exposure to bacteria and bacterial antigens. Their contribution to enteric mucosal immunity has been clearly evidenced in transgenic mice expressing the human Paneth cell α-defensin, HD-5 [39]. While HD-5 has direct antimicrobial activity against bacteria, HD-6 acts by creating nanonets that entrap bacteria and prevent further dissemination [40]. Paneth cells of mice also secrete their own α-defensins into the lumen of small intestinal crypts, and local concentrations have been estimated to be 25–100 mg/mL at the point of release [37]. Paneth cells were also shown to express LPLUNC1 which co-localizes with HD-5 in the secretory granules. LPLUNC1 is a protein similar to BPI which does not display antimicrobial activity *in vitro* but binds lipopolysaccharide (LPS) and inhibits the TLR4-signaling pathway in response to *V. cholerae* O1 LPS. LPLUNC1 mRNA is also the most highly up-regulated transcript in the small intestine during acute phase cholera [41].

***In fish***, epithelial defenses include a series of AMPs whose expression varies according to peptide families, fish species and tissues (for recent reviews see [42,43]). Indeed, fish AMPs are abundant in mucosal linings such as the skin, gills, and intestine, suggesting an important role in immunity [44]. These include AMPs similar to those found in mammals, namely β-defensins, cathelicidins, hepcidins and histone-derived AMPs together with α-helical peptides AMPs (pleurocidin, piscidins, and moronecidin, among others). Fish β-defensin genes have the highest basal expression in skin epithelium, which is induced by a variety of bacterial challenges such as *Aeromonas hydrophila* and *Vibrio anguillarum.* Interestingly, tissue-specific production of β-defensins has been described in salmonids where variants of this family can be differentially up-regulated in the intestine or gill tissues following bacterial challenge [45]. Hepcidin, which is both an AMP and a hormone expressed in liver, is also expressed by the skin epithelium and intestine. Fish hepcidin genes can be induced by exposure to both Gram-positive and Gram-negative bacteria. Cathelicidin is expressed in diverse epithelia including skin, gill and intestine. In the Atlantic cod, its expression in the gills was induced by *Aeromonas salmonicida* but not by *V. anguillarum* [46]. Like β-defensins, salmonid cathelicidins are produced in several mucosal tissues where variants display differential expression [47]. Moreover, transcripts of a homologue of the human bactericidal/permeability-increasing protein (BPI) have been found in the skin, intestine and gills of various fish species [48,49]. Finally, histone-derived AMPs are released in the epithelial mucosal layer of wounded fish skin [50]; they are expressed by mucus-producing globlet cells, the cells in which all the AMPs from fish skin accumulate [43].

***In marine invertebrates***, AMPs are also expressed by a broad range of epithelial cell types. Homologues of human BPI are produced by diverse invertebrate species. In the squid *Euprymna scolopes*, *Es-*LBP1 was found in the light organ of juvenile squids colonized by *V. fischeri*, but not in aposymbiotic squids. Expression was localized within the deep crypt spaces where the symbiotic vibrios reside and along the surface of the epithelia [51]. In the oyster *Crassostrea gigas*, a homologue of human BPI, *Cg*-BPI, is produced by various epithelial cell types including the intestine, gills, and mantle [52]. In addition, the *Cg*-Defm defensin is expressed by the oyster mantle, the shell-forming secretory epithelium [53]. Expression of *Cg*-BPI and *Cg*-Defm was constitutive in the epithelia of oysters infected with vibrios [14]. Recently, a novel AMP rich in lysine residues was extracted from oyster gills. This AMP called *Cg*-Molluscidin is predicted to form a α-helix [54]; its regulation in response to infection is still unknown. Moreover, as in fish, oyster epithelia accumulate histones displaying antimicrobial activity against vibrios [55]. These antimicrobial histones are released in response to infection or injury by infiltrating hemocytes, the circulating immune cells of the oyster, by a mechanism reminiscent of neutrophil extracellular traps in vertebrates [56].

**Table 2:** Antimicrobial peptides (AMPs) expressed in epithelial tissues.

Species	AMP family	Examples	Epithelial Tissues	References
Human	α-defensins	HD-5, HD-6	Small intestine, female genital tract	[37,57]
	β-defensins	hBD-1/-2/-3	Respiratory tract, large intestine, urogenital epithelium, oral cavity, skin	[58,59,60,61,62]
	Cathelicidins	LL-37(hCAP-18)	Skin, gastrointestinal tract, epididymis, lungs, oral cavity, ocular surface	[31,63,64], for review see [65]
	Bactericidal-permeability increasing proteins	BPI	Esophagus, respiratory tract, large intestine	For review see [66]
	C-type lectins	HIP/PAP	Small intestine	[36]
Fish	β-defensins	omDB-1/-2/-3/-4	Skin, gills, intestine	[45,67]
	Cathelicidins	rtCATH_1/-2A-2B, asCATH-1/-2 HFIAP-1/-2/-3	Skin, gills, intestine	[47,68]
	Liver-expressed antimicrobial peptides (LEAPs)	Hepcidin (LEAP-1), LEAP-2 Sal-1 Sal-2	Skin, intestine	[69], for review see [44]
	α-helical peptides	Pleurocidin, Piscidins Chrysophsins Moronecidin	Skin, gills	[70,71,72]
	Bactericidal-permeability increasing proteins	BPI	Intestine, gills	[48,49]
	Histone-derived AMPs	Parasin-1 Hipposin Oncorhyncin	Skin mucus	[73,74] [44,50]
Squid	LPS-binding/ Bactericidal-permeability increasing proteins	*Es-*LBP1	Light organ	[51]
Oyster	CS-αβ defensins	*Cg-*Defm	Mantle tissue	[53]
	Bactericidal-permeability increasing proteins	*Cg-*BPI	Gills, mantle, labial palps, gastrointestinal tract	[52]
	Histone-derived AMPs	*cv*H2B-1/-2/-3/-4	Gills	[55]
Coral	Cysteine Rich peptides	Damicornin Mytimacin-like	Oral ectoderm	[28]
	LPS-binding/ Bactericidal-permeability increasing proteins	LBP–BPI	Oral ectoderm	[28]

### 2.3. Vibrios Adapted to Intracellular Life in Phagocytes

Vibrios have traditionally been considered extracellular organisms. In recent years, however, vibrios (*V. cholerae* and *V. mimicus*) have been shown to also adopt intracellular stages in phagocytes from the environment, the amoebae [75,76,77] (Table 3). Similarly, live vibrios have been found inside professional phagocytes within the hosts they colonize. In vertebrates, *V. cholerae* can survive inside human macrophages; this intracellular stage is required for the T6SS-mediated secretion of factors causing actin cross-linking in host cells [5]. In invertebrates, a *V. splendidus*-related strain referred to as *V. tasmaniensis* LGP32 can survive in hemocytes, the circulating immune cells of the oyster (Table 3). Hemocyte invasion was accompanied by reduced production of reactive oxygen species and altered phagosome mutation [6]. While vibrios pathogenic for fish can adopt intracellular stages in epithelial cells [78,79], to our knowledge they have not been reported to invade professional phagocytes.

**Table 3:** Vibrios colonizing phagocytes.

Species or strain	Host cells	References
*V. cholerae* O1, O139	amoebae	[75,77]
*V. cholerae*	human macrophages	[5]
*V. mimicus*	amoebae	[76]
*V. tasmaniensis* LGP32	oyster hemocytes	[6]

### 2.4. AMPs of Phagocytes

Intracellular vibrios must face the potent chemical defenses of phagocytes, professional immune cells that circulate in the animal bloodstream and infiltrate infected tissues. Phagocyte defences include reactive oxygen species, which are particularly active during phagocytosis; hydrolytic enzymes including lysozyme; as well as AMPs, which are produced and stored by phagocytic cells (Table 4). 

Human phagocytes (neutrophils and macrophages) are indeed known to express a broad diversity of AMPs. Neutrophils express α-defensins, stored in azurophil granules that fuse with the phagolysosome to kill internalized bacteria, and the LL-37 cathelicidin, stored in secretory granules which release their content extracellularly. α-defensin expression is constitutive and their release is regulated by diverse microbial signals. In neutrophil phagolysosomes, the concentration of defensins has been estimated at ~10 mg/mL [80]. In addition, human neutrophils express the BPI antimicrobial protein [30]. In human macrophages, where *V. cholerae* is able to survive, AMPs such as LL-37, hepcidin and human β-defensin 1 and 2 can control intracellular pathogens [81,82]. Indeed, the crucial role of LL-37 in intracellular killing of mycobacteria has been extensively documented [83,84]. 

In fish, less information is available on AMPs expressed by phagocytes. AMPs of granulocytes include the α-helical peptide piscidin [85] and hepcidin in the seabream [86]. The BPI/LBP protein is constitutively expressed in head kidney leukocytes from Atlantic cod [49]. However, attention must be paid to the potential infiltration of phagocytes in tissues when AMPs expression is analyzed. Therefore, further studies are needed to determine whether the AMP expression in fish is restricted to a specific cell type or tissue. It is also not known whether fish phagocytes serve as a niche for any given *Vibrio* species.

Hemocytes of invertebrates also produce a large array of AMPs. Upon infection, oyster hemocytes massively migrate to infected tissues, bringing their antimicrobial content to the site of infection, and actively phagocytose bacteria. In oyster hemocytes, where the *V. tasmaniensis* strain LGP32 was found to survive, AMPs include defensins, big-defensins, proline-rich peptides, as well as a BPI antimicrobial protein (for review see [1]). BPI is stored in large cytoplasmic granules while the intracellular localization of the other AMPs is not yet known. Expression of BPI and big-defensin 1 and 2 is induced in hemocytes of infected oysters, whereas defensin expression is not regulated by the infection [14,87].

Similar to phagocytes from metazoans, amoebae, which can host diverse *Vibrio* species, produce pore-forming polypeptides such as the well-known amoebapores. These peptides are stored in cytoplasmic granules and can rapidly perforate human and bacterial cells. Amoebapores combat the growth of phagocytosed bacteria by permeabilizing their membranes inside the digestive vacuoles [88].

**Table 4:** AMPs expressed in phagocytes.

Species	AMP	Examples	Phagocytes	References
Human	α-defensins	HNP-1/-2/-3/-4	Neutrophils	[80]
	β-defensins	hBD-1/-2	Macrophages, Dendritic cells	[81,82]
	Cathelicidins	LL-37	Neutrophils	[89,90]
	Liver-expressed antimicrobial peptides (LEAPs)	Hepcidin	Granulocytes Macrophages	[91,92]
	Bactericidal-permeability increasing proteins	BPI	Neutrophils, (Eosinophils/to a lesser extent)	[30,93,94]
Fish	α-helical peptides	Piscidins	Granulocytes	[85]
	LPS-Binding/Bactericidal-permeability increasing proteins	LBP/BPI	Head–kidney leukocytes	[49]
Oyster	CS-αβ defensins	*Cg-*Defh-1/h2	Hemocytes	[1]
	Big-defensins	*Cg-*big-defensin-1/-2/-3	Hemocytes	[87]
	Proline-rich peptides	*Cg-*Prp	Hemocytes	[14]
	Bactericidal-permeability increasing protein	*Cg-*BPI	Hemocytes	[14,52]
	Histone-derived AMPs	H1- and H5-like histones	Hemocytes	[56]

## 3. Known Mechanisms of Resistance/Evasion to AMPs in Vibrios

### 3.1. Outer Membrane Remodeling

As electrostatic interactions often play a crucial role in the initial interaction of cationic AMPs with bacterial membranes, both Gram-negative and Gram-positive bacteria have evolved strategies to neutralize the net negative charge of cell surface molecules with amine-containing substitutions. Thus, D-alanylation of teichoic acids, which are major components of the Gram-positive cell wall, confers AMP-resistance in a variety of Gram-positive bacteria including *Staphylococcus aureus* and *Bacillus cereus* [95,96]. More generally, aminoacylation of bacterial cell surface phosphatidylglycerols with L-lysine, L-alanine, or D-alanine confers resistance to cationic AMPs in both Gram-positive and Gram-negative bacteria [97].

**Figure 1:**
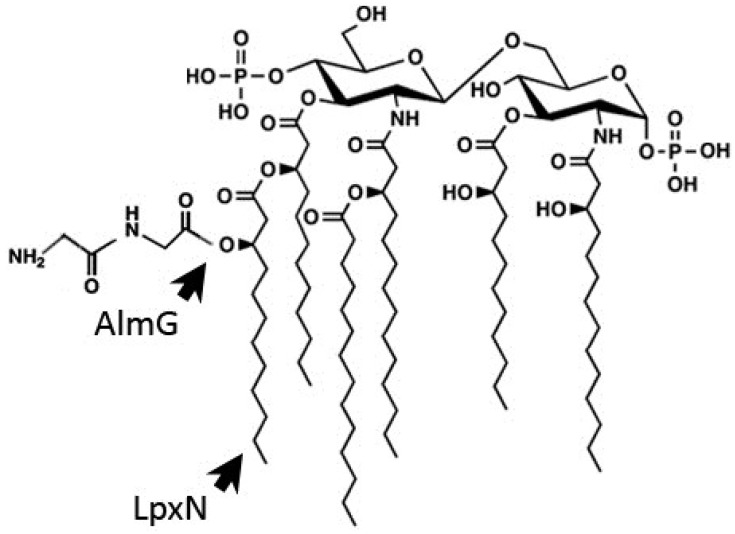
Structure of modified lipid A from *V. cholerae* O1 El Tor. The structure of *V. cholerae* lipid A was established by Hankins *et al.* (2011) [99]. It is composed of a β 1′-6 linked glucosamine disaccharide with unmodified 1- and 4′-phosphate groups, which is acylated at the 2-, 3-, 2′- and 3′-positions. Myristate (C14:0) and 3-hydroxylaurate (3-OH C12:0) are ester-linked to the hydroxyl groups on the 2′- and 3′-linked fatty acyl chains. The 3-hydroxylaurate secondary acyl chain transferred by the LpxN acyltransferase is required for AMP resistance. Similarly, the di-Glycine residues transferred by the AlmG to the hexa-acylated lipid A of *V. cholerae* O1 El Tor strains are crucial for AMP-resistance [101].

LPS, the major constituent in the Gram-negative outer membrane, is composed of three regions: the anionic Lipid A membrane anchor, the core oligosaccharide and the O-antigen polysaccharide [98]. Hankins *et al.* have shown that *V. cholerae* O1 and O139 share identical asymmetrical hexa-acylated Lipid A structures [99] composed of a β 1'-6 linked glucosamine disaccharide with unmodified 1- and 4'-phosphate groups, which is acylated at the 2-, 3-, 2'- and 3'-positions. Myristate (C14:0) and 3-hydroxylaurate (3-OH C12:0) are ester-linked to the hydroxyl groups on the 2'- and 3'-linked fatty acyl chains (Figure 1). As in *V. cholerae*, the presence of a hydroxylated secondary acyl chain has been reported in the Lipid A structure of *V. fischeri* [100].

Polymyxin B (PmB) has been extensively used to study the molecular basis of bacterial resistance to cationic AMPs in Gram-negative bacteria. Indeed, this peptide produced by the Gram-positive *Paenibacillus polymyxa* disrupts the cell envelope of Gram-negative bacteria by associating with the anionic LPS as well as with acidic glycerophospholipids [102]. To resist to AMPs, Gram-negative bacteria can neutralize their cell membrane by transferring phosphoethanolamine or aminoarabinose to phosphate groups on the lipid A domain of LPS [103].

In *V. cholerae*, the secondary acyltransferase VC0212 (LpxN or MsbB), which transfers a 3-hydroxylaurate group to penta-acylated Lipid A, contributes to the resistance of an El Tor strain to AMPs including PmB and LL-37 [99,104]. Thus, the higher susceptibility of the *vc0212* mutant displaying incomplete Lipid A might be due to the greater permeability of its bacterial membrane. However, recent data by Hankins *et al*. demonstrated that the presence of a 3-hydroxyl group on the secondary acyl chain provides a site for esterification of glycine residues in a unique strategy necessary for resistance to PmB in *V. cholerae* [101] (Figure 1). 

Three *V. cholerae* proteins, VC1577 (AlmG), VC1578 (AlmF), and VC1579 (AlmE) sharing sequence homology with the machinery involved in D-alanylation of teichoic acids in Gram-positive bacteria are essential for Lipid A modification with glycine and diglycine residues through aminoacyl esterification (Figure 1). Interestingly, sequence alignments comparing the classical (susceptible to PmB) and the El Tor (resistant to PmB) biotypes of *V. cholerae* revealed that the classical strain O395 has a nonsense mutation, resulting in a truncated AlmF carrier protein lacking the conserved serine [101]. The authors discovered that classical strains lack glycine-modified Lipid A. Upon *alm* mutation, the minimum inhibitory concentration (MIC) of PmB against El Tor strains dropped dramatically (~100 times) from 96–128 μg/mL to 0.5–1.0 μg/mL, showing that glycine modification of Lipid A is an essential mechanism of AMP resistance in *V. cholerae*. This study provides a well-defined mechanism for the different PmB-resistant phenotypes observed in *V. cholerae* classical and El Tor biotypes. Why classical strains appear to have lost carrier protein functionality and thus AMP resistance is a puzzling evolutionary question.

To date, it is unknown whether modifications of *Vibrio* LPS are induced upon exposure to sublethal concentrations of cationic AMPs, as shown in other bacterial species like Salmonella Typhimurium, which regulate their LPS structure, contributing to resistance to cationic AMP [105]. Changes in *Salmonella* LPS structure, regulated by the two-component system PhoPQ, include reducing average O-antigen chain-length, acylating, deacylating, and hydroxylating lipid A, derivatizing lipid A and LPS core phosphates with cationic groups (for recent review see [106]). Homologues of PhoPQ are found in *Vibrio* species, however, the potential role of PhoPQ in resistance to AMPs has not been described to date.

### 3.2. Induction of the Envelope Stress Response

As discussed above, many AMPs create damage to bacterial membranes as part of their mechanism of action. Sensing external stress is therefore crucial to combating membrane injury before the damage becomes irreversible. One of the strategies by which bacteria respond to outer membrane stress and modulate gene expression is via the alternate σE factor, encoded by the *rpoE* gene. Under non-stress conditions, σE is inactivated by its cognate anti-sigma factor localized to the inner membrane. When activated by envelope stress, *i.e.*, misfolding of outer membrane proteins, σE promotes the expression of factors that help preserve and/or restore cell envelope integrity. Certain outer membrane proteins can serve as upstream signal sensors to modulate the activity of σE [107]. In *V. cholerae,* the major outer membrane OmpU is a key determinant of σE production [108]. Such dependence on a single factor contrasts with the regulation of σE in *E. coli*, in which numerous factors contribute to its activation and none is dominant. 

In *V. cholerae*, σE plays a role in outer membrane stress response and resistance to AMPs. Thus, deficiency of σE confers to *V. cholerae* greater sensitivity to the antimicrobial peptide P2, a synthetic derivative of human BPI. Consistent with the *ompU*-dependent activation of σE, lack of OmpU in *V. cholerae* also conferred a greater sensitivity to AMPs [109,110]. Similar results were obtained for the oyster pathogen *V. tasmaniensis* LGP32 in which OmpU contributed to resistance to the oyster antimicrobials *Cg*-Defm and *Cg-*BPI [111]. However, in both *V. cholerae* and *V. tasmaniensis*, OmpU-mediated resistance was much lower than that conferred by Lipid A remodeling [101]. Moreover, in *V. tasmaniensis* LGP32, the major negative effect of the *ompU* deletion on pathogenicity was attributed to impaired capacity to invade the oyster immune cells rather than lower resistance to oyster AMPs [6].

### 3.3. AMP Titration by Outer Membrane Vesicles

One σE-dependent mechanism whose role in AMP resistance has been less studied is outer membrane vesicle release. OMVs form the insoluble fraction of Gram-negative bacteria extracellular products; they are extruded from the bacterial cell surface and entrap some of the underlying periplasmic contents [112,113]. OMVs are key players in the interaction between Gram-negative bacteria and both the prokaryotic and eukaryotic cells from their environment [114]. Whereas it is now well established that *Vibrio* spp. constitutively release OMVs during cell growth [115,116,117], only recent studies in *E. coli* [118] and *Vibrio* spp. [119,120] have shown that the release of OMVs protects bacteria against membrane-active AMPs.

In *V. cholerae*, earlier work demonstrated that under envelope stress conditions, the small regulatory RNA VrrA is expressed in a σE-dependent manner to down-regulate OmpA, which in turn reduces envelope stress by promoting OMV release [121]. More recently, we found that physiologically relevant amounts of OMVs produced in the presence of a sub-lethal concentration of PmB provide protection against human cathelicidin LL-37, increasing the MIC of LL-37 by four-fold. This cross-protection has been attributed to the presence of the biofilm-associated extracellular matrix protein Bap1, which is associated with OMVs in larger amounts when bacteria are grown in the presence of PmB. The Bap1 protein can therefore trap LL-37, leading to increased resistance of *V. cholerae* towards LL-37 [119].

**Figure 2:**
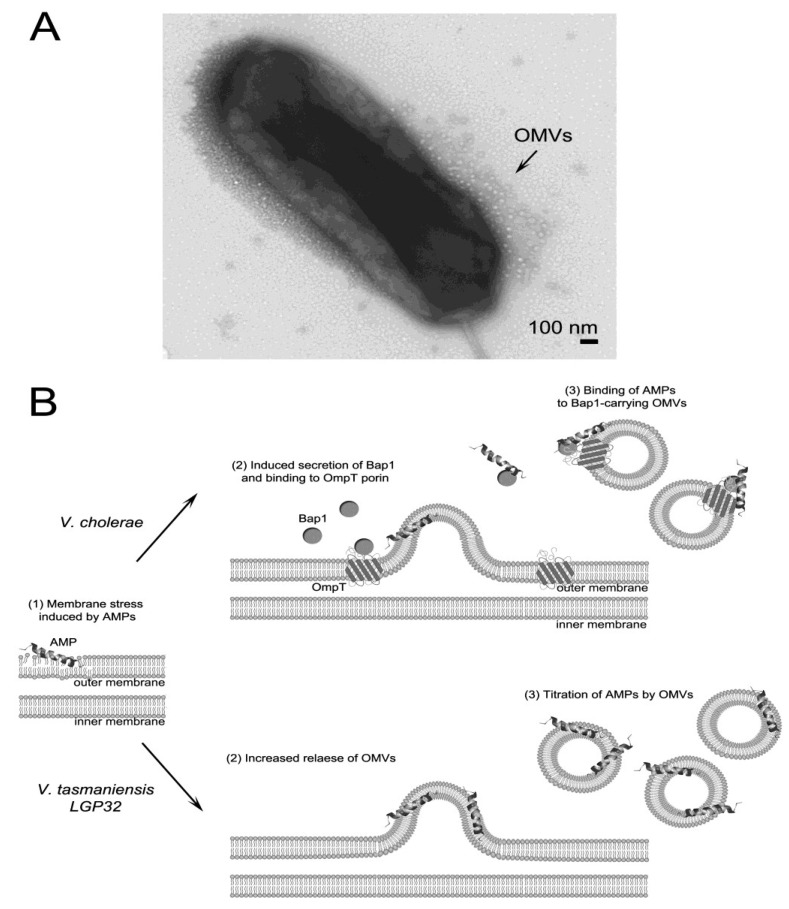
Model for AMP-titration by outer membrane vesicles (OMVs) in *V. cholerae* and *V. tasmaniensis*. (**A**) OMVs released in the extracellular medium by the hypervesiculating Δ*ompU* mutant of *V. tasmaniensis* strain LGP32. Logarithmic phase cultures were negatively stained and observed by transmission electron microscopy as described in [120]; (**B)** The role of OMVs in the protection against AMPs has been recently described in two species of vibrios. In *V. cholerae*, OMVs cross-protect against the human cathelicidin LL-37 when bacteria are exposed to sublethal concentrations of PmB. Those OMVs are associated with Bap1 protein which serves as a ligand for LL-37. The association of Bap1 to OMVs is mediated by the outer membrane protein, OmpT [119]. In *V. tasmaniensis*, OMVs produced in the absence of AMPs are sufficient to titrate PmB and confer a potent dose-dependent protection against PmB. Although the molecular basis of PmB binding to *V. tasmaniensis* OMVs remain unknown, it is speculated that titration may occur by PmB insertion in the OMV membranes. The release of OMVs was shown to be strongly enhanced by the contact of *V. tasmaniensis* with oyster plasma [120]. In both species, OMV release is thought to create a protective membranous shield that prevents the interaction of membrane-active AMPs with the bacterial membranes.

In *V. tasmaniensis* LGP32, OMVs provide significant and dose-dependent protection against AMPs [120]. Indeed, OMVs increased the MIC of PmB from 2–16-fold at OMV concentrations ranging from 6.25–50 μg/mL. This protective effect was attributed to the binding of PmB to OMVs; no proteolytic degradation of the peptide was observed. Interestingly, the addition of oyster plasma to the culture medium strongly stimulated the release of OMVs by *V. tasmaniensis* LGP32. This indicates that as in *E. coli,* in which sub-lethal concentrations of AMPs promote OMV release [118], OMV release in vibrios is likely up-regulated by membrane-active agents in oyster plasma. Consistent with this hypothesis, LGP32 lacking the major outer membrane protein OmpU, which controls envelope stress signaling in vibrios [108], showed a hypervesiculation phenotype (Figure 2A).

Altogether, these recent studies indicate that OMVs are a potent strategy used by vibrios to trap membrane-active AMPs such as PmB or LL-37, forming a protective shield that prevents interaction with the membranes of the bacterial cell (Figure 2B). Although OMVs released by vibrios can contain specific proteases like the recently identified vesicular serine protease Vsp (VS_II0815) of *V. tasmaniensis* LGP32, there is to date no evidence of AMP degradation by OMV-encapsulated content [119,120].

### 3.4. Efflux of AMPs

The involvement of efflux pumps in antimicrobial resistance, especially in antibiotic resistance, is well established in Gram-negative bacteria [122,123]. There are five different active efflux systems described in bacteria: the ATP-binding cassette superfamily (ABC), the small multidrug resistance family (SMR), the multi antimicrobial extrusion protein family (MATE), the major facilitator superfamily (MFS), and the resistance-nodulation-cell division superfamily (RND) [124]. In terms of antimicrobial resistance, the RND family efflux pumps are particularly important in Gram-negative bacteria. RND efflux systems are composed of an outer membrane protein homologous to the transmembrane β-barrel TolC protein of *E. coli*, a periplasmic membrane fusion protein (MFP), and an integral cytoplasmic membrane pump protein belonging to the RND superfamily of transporters (for review see [125]). These three components function to form a channel to extrude substrates from the cell envelope into the environment. The *V. cholerae* VexAB-TolC [126,127], the *E. coli* and *Salmonella enterica* AcrAB-TolC [128,129], and the *Pseudomonas aeruginosa* MexAB-OprM systems [130] function as RND efflux systems. 

In *V. cholerae*, six RND efflux pumps have been described: VexAB, VexCD, VexEF, VexGH, VexIJK, and VexLM [131]. Among them, four are required for antimicrobial resistance *in vitro.* VexAB is the main efflux pump involved in the resistance to antimicrobials including bile acids, detergents, antibiotics, and PmB. The MIC of PmB dropped by four-fold (from 110–27 μg/mL) after *vexB* mutation in *V. cholerae* [127,132]. Moreover, the MIC of PmB against the *vexB* mutant was comparable with the MIC against the RND-null strain, indicating that only VexAB is involved in resistance to PmB [127]. Besides VexAB, VexGH also contributes to antibiotic (novobiocin and ampicillin) and detergent resistance but to a lesser extent than VexAB. Indeed, a decrease in the MIC can be observed only for a *vexBH* double mutant but not for the *vexH* single mutant, compared to the wild-type and *vexB* single mutant strains [133]. Finally, VexCD and VexIJK appeared to efflux bile acids and detergents, respectively [127,132]. VexEF and VexLM do not participate in antimicrobial resistance, but are required for the full virulence of *V. cholerae* by influencing the production of the major effectors of virulence, *i.e.*, cholera toxin and the toxin co-regulated pilus [133]. In *V. parahaemolyticus*, proteomic identification of membrane proteins up-regulated in strains that artificially evolved resistant to AMPs, (including the fish AMP pleurocidin) led to the identification of TolC [134]. Unfortunately, its role in AMP resistance in *V. parahaemolyticus* has not been investigated further*.*

In addition to efflux pumps, a K^+^ pump encoded by the *trkA* gene has been described in *V. vulnificus*, and its role in AMP and serum resistance investigated [135]. The *trkA* gene product, TrkA, is a cytoplasmic protein bound to the inner side of the cytoplasmic membrane [136]. In *V. vulnificus*, the *trkA* mutant exhibited attenuated growth at intermediate potassium concentrations and was more sensitive to human serum protamine and PmB than was the wild type. Indeed, in contrast to the wild-type strain, the *trkA* mutant lysed in the presence of 10–20 μg/mL of protamine, and 5–15 μg/mL of PmB [135]. Moreover, TrkA was found to be important for *V. vulnificus* virulence in mice [135].

### 3.5. Suppression of AMP Expression

Pathogenic bacteria have developed multiple modalities to combat the antimicrobial response of their hosts. In addition to the structural modifications reviewed above, which increase their resistance to AMPs, they also use transcriptional repression as a strategy to evade the host immune system. Thus, the down-regulation of AMPs can be considered a general mechanism to facilitate invasion of pathogenic bacteria, including vibrios. 

In humans, where the interaction of *V. cholerae* with intestinal epithelial cells is a critical step in the disease process, down-regulation of the cathelicidin LL-37, but not of the defensin HBD-1 has been reported in the presence of enteric pathogens including *V. cholerae* O139 [137]. The authors showed that cholera toxin (CT) was the predominant molecule associated with the regulation of AMPs by *V. cholerae* spp. *in vitro* and *in vivo* using intestinal epithelial cells and ileal loop experiments, respectively [137]. Moreover, multiple signaling pathways activated downstream of intracellular accumulation of cAMP contribute to the CT-mediated suppression of LL-37 in intestinal epithelial cells [137]. However, a more recent study on small intestine biopsies of patients with *V. cholerae* O1 infections did not show transcriptional repression of AMP genes in the small intestine [138], a discrepancy that might be explained by differences in transcriptional regulation *in vivo* and *in vitro*. *In vivo*, the expression of hBD-1, -3 and -4 did not vary with the infection, whereas hBD-2 mRNA levels were significantly higher at the acute stage of cholera than at the convalescent stage and in healthy controls. Paneth cell-derived HD-5 and HD-6, which were all expressed at high levels in controls, were not affected by the infections. While no transcriptional repression could be observed, the authors reported that hBD-2, HD-5 and LL-37 peptides are normally present in the small intestine epithelium and amounts decrease at the acute stage of watery diarrhea. Lower amounts of HD-5 could result from degranulation of the Paneth cells in response to infection. The processes regulating hBD-2 and LL-7 levels remain to be characterized.

In invertebrate hosts, similar downregulation of antimicrobial peptides and proteins has been observed during vibrioses. For instance, the coral pathogen, *V. coralliilyticus*, represses the expression of the damicornin, an AMP expressed by the scleractinian coral *Pocillopora damicornis* [28]. Indeed, damicornin transcripts increased during the first 6 days after infection with *V. coralliilyticus*, directly followed by a dramatic decrease from days 9–18. Conversely, no transcriptional change was observed when *P. damicornis* was exposed to a nonvirulent state of *V. coralliilyticus* [28]. Since *V. coralliilyticus* enters into the ectodermal coral tissue within 6 days, the authors suggested that a first phase of infection, involving bacterial recognition by host cells, triggers a nonspecific inflammatory response that activates damicornin gene transcription. In a second phase, following bacterial invasion, the pathogen suppresses damicornin transcription. This study represents the first characterization of the immunosuppression of AMP expression in an invertebrate-vibrio model of pathogenesis. More recently, using a global RNAseq approach, the same authors showed that not only damicornin, but also a mytimacin-like and a LBP-BPI gene displayed decreased expression during a successful *V. coralliilyticus* infection [139].

In mollusks, repression of AMP transcription has not been demonstrated *per se*. Indeed, upon infection of oysters with the pathogen *V. tasmaniensis* LGP32, major hemocyte movements occur which, by bringing AMP-producing hemocytes to infected tissues, create an apparent depletion in *Cg*-Defm and *Cg*-BPI transcripts in the circulating hemocytes. However, those transcripts accumulate at the same time in the hemocyte-infiltrated tissues [14]. A similar apparent repression of defensin expression was observed in the circulating hemocytes of a heterologous host, the mussel, infected with *V. tasmaniensis* LGP32 [140]. However, to date, the only AMP whose transcription is likely down-regulated by LGP32 is a proline-rich peptide from the oyster which acts by synergism with the other AMPs [14]. 

## 4. Conclusions

While vibrios have evolved the capacity to colonize immune tissues such as epithelia and phagocytes, only recent studies have started to investigate the mechanism by which they can survive the high AMP concentrations they encounter. Among their potent mechanisms of resistance to AMPs, vibrios use novel mechanisms of membrane remodeling. In particular, some highly resistant strains substitute their hexaacylated Lipid A with a diglycine residue to reduce the negative charge of their surface thereby lowering the electrostatic interaction with cationic AMPs. As a response to envelope stress, which can be induced by membrane-active agents including AMPs, vibrios release outer membrane vesicles to create a protective membranous shield that traps AMPs and prevents interaction of the peptides with their own membranes. Finally, once AMPs have breached the bacterial membrane barriers, vibrios can use RND pumps similar to those of other species to transport AMPs out of their cytoplasmic space. Although suppression of AMP transcription has been described in some host–pathogen interactions, this mechanism of immune evasion appears to be more specific to given strains/species than universal among vibrios.
